# Factors that cause women with osteoporosis to fall

**DOI:** 10.20945/2359-3997000000578

**Published:** 2023-05-10

**Authors:** Renata Gonçalves Pinheiro Correa, Madeline Luiza Ferreira Pivovarsky, Guilherme da Silva Santos, Anna Raquel Silveira Gomes, Victoria Zeghbi Cochenski Borba

**Affiliations:** 1 Universidade Federal do Paraná Programa de Pós-graduação em Medicina Interna e Ciências da Saúde Curitiba PR Brasil Programa de Pós-graduação em Medicina Interna e Ciências da Saúde da Universidade Federal do Paraná, Curitiba, PR, Brasil; 2 Universidade Federal do Paraná Programa de Pós-graduação em Educação Física Curitiba PR Brasil Programa de Pós-graduação em Educação Física, Universidade Federal do Paraná, Curitiba, PR, Brasil; 3 Universidade Federal do Paraná Faculdade de Medicina Curitiba PR Brasil Faculdade de Medicina, Universidade Federal do Paraná, Curitiba, PR, Brasil; 4 Universidade Federal do Paraná Programa de Pós-graduação em Educação Física Departamento de Fisioterapia Curitiba PR Brasil Departamento de Fisioterapia, Programa de Pós-graduação em Educação Física, Universidade Federal do Paraná, Curitiba, PR, Brasil; 5 Universidade Federal do Paraná Divisão de Endocrinologia Departamento de Medicina Interna Curitiba PR Brasil Departamento de Medicina Interna, Divisão de Endocrinologia (SEMPR), Universidade Federal do Paraná, Curitiba, PR, Brasil

**Keywords:** Elderly, osteoporosis, falls, intrinsic factors, extrinsic factors

## Abstract

**Objectives:**

To analyze and compare intrinsic and extrinsic factors that cause falls among women receiving treatment for osteoporosis.

**Subjects and methods:**

A cross-sectional study of women ≥50 years receiving treatment for osteoporosis. Participants filled out questionnaires (demographic characteristics), and researchers took anthropometric measurements of bone mineral density, handgrip strength (HGS), ankle range of motion (ROM), and gait speed (GS). We also evaluated the Timed Up and Go Test (TUGT), Five Times Sit-to-Stand Test (SST), and Falls Efficacy Scale–International (FES-I) and investigated the extrinsic factors for falls.

**Results:**

We included 144 participants (71.6 [8.3 years]), who reported 133 falls. We classified participants into a non-faller group (NFG; 0 falls, n = 71, 49.5%), a faller group (FG; 1 fall, n = 42, 28.9%), and a recurrent-faller group (RFG; more than 1 fall, n = 31, 21.5%). Most patients had an increased risk of falling according to the TUGT, SST, reduced ankle ROM, and GS (P < .005 for all). FES-I was associated with sporadic and recurrent falls. For the multivariate analysis, the number of falls was influenced by the presence of ramps (RR 0.48, 95% CI, 0.26-0.87, P = .015), uneven surfaces (RR 1.6, 95% CI. 1.05-2.43, P = .028), and antislippery adhesive on stairs (RR 2.75, 95% CI, 1.77-4.28, P < .001).

**Conclusion:**

Patients receiving treatment for osteoporosis are influenced by intrinsic and extrinsic factors that cause falls. Lower-limb strength and power-discriminated participants at a higher risk of falls, but extrinsic factors varied. Only uneven floors and antislippery adhesives on stairs were associated with increased frequency of falls.

## INTRODUCTION

Elderly women have a high prevalence of osteoporosis (30%), which increases with age. It reaches 42% by the age of 70, which can lead to negative outcomes. such as decreased strength, functional capacity, and balance, increasing falls and fractures ( 1 , 2 ). Falls cause 87% of all fractures in elderly women with osteoporosis, usually due to low-impact trauma ( 1 , 2 ). Guidelines from the American and British Geriatrics Societies recommend annual screening for risk of falls in people age 65 years and over, which is an important measure to prevent falls and guide appropriate interventions ( 2 ).

Falls are considered a public health problem and affect elderly people with multiple comorbidities, including osteoporosis. A fall is defined as any unintentional change in the position of the body that results in unexpected contact with the ground or any other surface close to the ground ( 3 ). Several intrinsic and extrinsic factors can lead to falls, and most are home related ( 4 ). Intrinsic factors for falls include declines in various functions inherent to the individual, such as limb muscle strength and power, vision, cognition, balance, and ankle range of motion (ROM). Ankle ROM is important because it helps people clear obstacles, decreasing the frequency of trips and falls. Psychological factors, such as fear of falls and depression, can also contribute to falls ( 3 , 4 ). Extrinsic factors for falls include external influences on the individual, such as the use of medications, conditions related to footwear, assistant devices, and domestic environmental or social conditions that favor tripping or slipping ( 5 ).

Some factors that contribute to fall risk (eg, age, sex, and history of previous falls) cannot be modified, but others can (eg, poor muscle strength, impaired balance or vision, vitamin D insufficiency, use of psychotropic medications or sedatives, and environmental/home fall hazards) and should be addressed ( 6 ). For this reason, a multifactorial approach to prevent falls is recommended for the elderly, including home environmental risks assessments ( 7 ). Low bone mineral density (BMD) influences falls and fractures due to functional decline, poor quality of life, and high fear of falling, which puts patients with osteoporosis at greater risk of falls and recommends self-limitation in an attempt to prevent further falls ( 7 , 8 ).

Although risk factors for falls are well documented in elderly populations, few studies have analyzed the difference between intrinsic and extrinsic factors between fallers and nonfallers in postmenopausal women with low BMD. In these studies, researchers investigated the risk of falls only based on clinical data on fractures and bone densitometry without accounting for the frequency of falls ( 8 , 9 ).

Therefore, the aim of this study was to conduct analyses and comparisons to determine whether specific intrinsic and extrinsic factors were related to the frequency of falls in postmenopausal women being treated for osteoporosis.

## SUBJECTS AND METHODS

### Study design

Cross-sectional, observational study approved by the research ethics committee of the *Hospital de Clínicas* at *Universidade Federal do Paraná* with protocol number 3320592 (CAAE: 02897818.6.0000.0096). All participants signed an informed-consent form. We conducted the study from November 2018 to March 2020, and we describe the results according to the Strengthening the Reporting of Observational Studies in Epidemiology (STROBE) guidelines. They are in agreement with the Declaration of Helsinki.

The study included community-dwelling women age 50 and older who were functionally independent, able to perform the required study tests, and receiving treatment for osteoporosis at the outpatient clinic of the Endocrinology and Metabology Service of *Hospital de Clínicas* at *Universidade Federal do Paraná* (SEMPR). We excluded women younger than 50 years who had neurological and/or orthopedic disorders, used prostheses, had metallic or nonmetallic implants, or suffered from decompensated chronic diseases at the time of the evaluation. We selected the patients by convenience during their routine appointment at the outpatient clinic. While they waited for their appointments, we verbally invited all of the patients in the waiting room to participate in the study. We evaluated those who accepted at the clinic on the same day of the appointment. We extracted data that we could not obtain during the evaluation from the participants’ medical records. We initially asked the participants to answer questions about demographics, lifestyle habits (eg, calcium intake from dairy products, physical activity, and smoking and drinking habits), medical history, and number of falls and they answered the questionnaires. Following this initial assessment, we measured the participants’ anthropometric and calf circumference (CC); tested them for handgrip strength (HGS), ankle ROM, and 4-m gait speed (GS); and evaluated them using the Five Times Sit-to-Stand Test (SST), Mini-Mental State Examination (MMSE), Timed Up and Go Test (TUGT), and BMD. We thoroughly questioned all patients using a list of extrinsic risk factors for falls and safety factors against falls. Trained researchers conducted the evaluations (ie, physical educator professionals, physical therapists, nutritionists, and physicians).

### History of falls

We defined falls as any event resulting in body change that causes an individual to fall inadvertently to the ground. This does not include falls resulting from a violent blow, sudden paralysis, loss of consciousness, or seizure ( 10 , 11 ). The history of falls was self-reported and evaluated with the question, “Have you experienced any falls within the last 12 months? If so, how many times?”

Based on their history of falls, we divided the participants into 3 groups: nonfallers (NFGs), when reporting no falls; fallers (FGs), when reporting a single fall; and recurrent fallers (RFGs), when reporting 2 or more falls in the previous year ( 11 ).

### Anthropometric assessment and cognitive screening

We measured weight using a calibrated anthropometric mechanical scale (Plenna^®^) with the participant barefoot and only wearing underwear. We measured height using a vertical anthropometer (Tonelli Gomes^®^). We calculated body mass index (BMI) by dividing the participant's weight (in kg) by their squared height (in m). We classified the BMI results as malnutrition (BMI <18.5 kg/m^2^) ( 12 ), normal (23 kg/m^2^ < BMI < 28 kg/m^2^), preobesity (28 kg/m^2^ < BMI < 30 kg/m^2^), and obesity (BMI > 30 kg/m^2^) ( 13 ). We measured waist circumference (WC) using inelastic tape placed at the midpoint between the anterior iliac crest and the lowest rib, with the patient standing up and at the end of expiration. We considered it normal when it was <88 cm ( 14 ).

We assessed cognition using the MMSE. The cutoff scores for detection of cognitive disorders were 18/19 for uneducated individuals and 24/25 for educated individuals ( 15 ).

### Bone mineral density

We assessed the participants’ BMD using dual-energy X-ray absorptiometry (DXA) on a Lunar Prodigy (serial number PA+302284, GE Medical Systems, Madison, WI, USA) or Horizon A (serial number 201383, Hologic, Bedford, MA, USA) device. All assessments followed the recommendations of the International Society for Clinical Densitometry (ISCD) for equipment maintenance and calibration. We obtained spine and hip (neck and total) BMD results closest to the study evaluation from the participants’ medical records. We classified the results according to the World Health Organization (WHO) and ISCD criteria as normal (T-score ≥-1.0 standard deviation [SD]), osteopenia (T-score between −1.1 and −2.49 SDs), and osteoporosis (T-score ≤-2.5 SDs) (ISCD; WHO, 2012).

### Assessment of intrinsic factors related to falls

The TUGT evaluated functional mobility and risk of falls. We conducted the test twice, the first time for a practice trial and the second for time for recording. A physical therapist guided the participants with the following verbal command: “ *Stay seated, and at the command of ‘go’, please stand up from the chair and walk at a comfortable and safe pace until you reach that cone 3 meters from here. Turn around at that line, go back to your chair, and sit down again.”* The instructor stopped the timer when the participant sat down again (ie, when the participant's trunk rested against the back of the chair) [ 16 ). The following cutoff values indicated reduced functional mobility and risk of falls according to the participants’ age: 60-69 years (>8.1 seconds [s]), 70-79 years (>9.2 s), ≥ 80 years (>11.3 s) ( 17 ).

The GS assessment recorded the time required for the participant to walk 4 m. For this test, we marked a distance of 4 m on the floor using masking tape. We positioned the participant at the 0-m mark and gave the following command: *“Walk at your usual pace, as if you were walking on the street. When I say ‘ready,’ start walking.”* We calculated the result by dividing the distance (4 m) by the time required to walk that distance (in s), and presented speed in meters per second (m/s). We conducted two iterations, one for a practice trial and the second for time recording. Based on the cutoff values, we considered speeds ≤0.6 m/s low, 0.6-1 m/s moderately low, 1-1.3 m/s normal, and ≥ 1.3 m/s fast ( 17 ).

The HGS assessment measured muscle strength using the Hydraulic Hand Dynamometer (Saehan SH5001^®^). The participant was seated on a chair without an armrest, with feet on the ground and hips and knees at 90° flexion. The participants’ shoulders were positioned in adduction and neutral rotation and their elbows at 90° flexion with the forearm and wrist in a neutral position. We instructed the participant to perform the maximum handgrip movement of the dominant upper limb for 3 s, performing 3 maximum movements with 1-2 minutes of rest between each repetition. We recorded the highest result obtained from 3 attempts for each participant (in kilogram-force [kgf]). We individually adjusted the dynamometer's grip based on the size of the participants’ hands. A result below 16 kg indicated low muscle strength ( 17 ).

The SST assessment evaluated lower-limb strength and power. We instructed the participants to stand up and sit 5 five times as quickly as possible on a 43-cm-tall chair while keeping their arms folded across their chest (ie, without help from their arms). Time recording (in s) started at the command “Now” and stopped when the participant sat down for the 5th time. We conducted the test 3 times, with an interval of 1 minute between each repetition. We calculated the result as the average of the 3 trials. We used a 15-s cutoff value to assess the risk of recurrent falls. We established the cutoff values for falls according to the participants’ age: 60-69 years (>11.4 s), 70-79 years (>12.6 s), and 80-89 years (>12.7 s) ( 17 , 18 ).

### Assessment of muscle mass

We measured muscle mass with CC measurement using a measuring tape around the largest diameter of the dominant leg's calf. The participant remained standing, with feet about 20 cm apart and weight distributed equally on both legs. Results below 31 cm indicated muscle mass depletion ( 17 , 18 ).

### Assessment of ankle joint mobility

We evaluated ankle ROM during plantar flexion and dorsiflexion movements performed actively and without previous heating with a goniometer (Carci^®^) positioned with the axis below the lateral malleolus, with the fixed arm aligned with the participant's fibular head and the movable arm parallel to the foot's lateral edge. We performed an average of 3 repetitions of each joint movement and expressed the ROM in degrees ( 19 ). The cutoff values of 26 (6.3)° for dorsiflexion and 57 (7.2)° for plantar flexion indicated decreased mobility ( 19 ).

### Fear of falling

We assessed fear of falling using the Falls Efficacy Scale – International (FES-I-BRASIL) questionnaire translated into Brazilian Portuguese and validated for the Brazilian population. This scale evaluates the participant's concern about the possibility of falling when performing 16 activities, each with scores ranging from 1 to 4 points. Scores ≥23 are associated with a history of sporadic falls and >31 points with that of recurrent falls ( 20 ).

### Assessment of extrinsic factors related to falls

To evaluate the presence of extrinsic factors for falls at home, we asked the participants about the presence of accessories increasing the risk of falls and improving safety against falls in their homes. We considered the following risk factors: presence of stairs, slippery bathroom floor, loose pets, uneven surfaces (obstacles that has to be overcome), slippery surfaces, ramps, dim lighting (causing vision problems), obstacles and tripping hazards (loose rugs or floor mats), missing tiles, loose cables or wires (extensions), and inappropriate bed height. Safety accessories included antislip adhesives on the stairs, grab bars in the bathroom, handrails on stairs, antislip adhesives on ramps, handrail on ramps, antislip adhesives for rugs, tall chairs, and tall toilets.

### Statistical analysis

We present the results as mean (SD). We used the Kolmogorov-Smirnov test to verify the normality of the variables’ distribution. To analyze differences between groups, we conducted one-way analysis of variance (ANOVA) (parametric) and Kruskal-Wallis (non-parametric) tests, depending on the variables’ normality. We used Bonferroni for post hoc testing. For multivariate analysis, we used the negative binomial regression model, and we present the resulting relative risks with 95% confidence intervals (95% CIs) and *P* values.

We conducted all analyses using the Statistical Package for the Social Sciences (SPSS) version 25. The significance level adopted was *P* < .05.

## RESULTS

**Figure 1:**
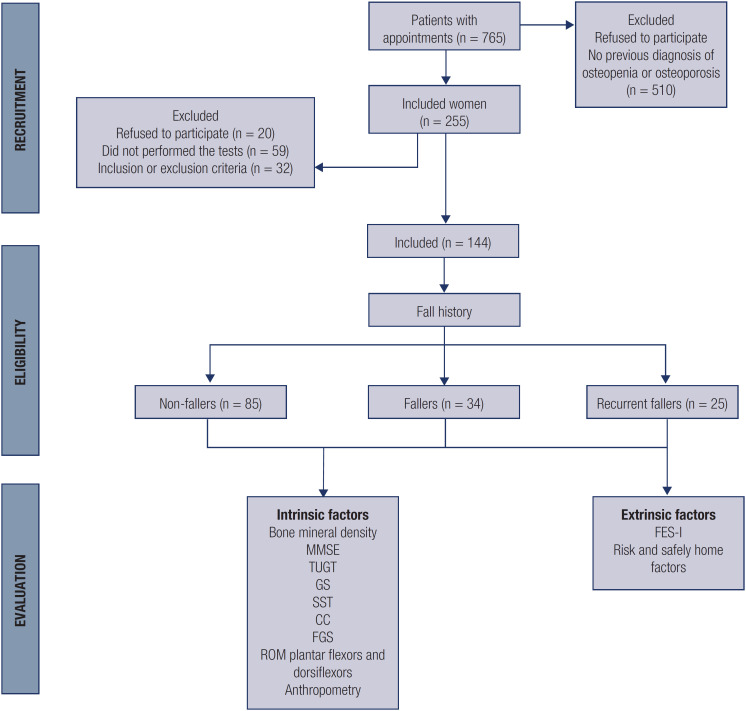
Study flowchart.

MMSE: Mini-Mental State Examination; TUGT: Timed Up and Go Test; GS: gait speed; SST: Sit-to-Stand Test; CC: calf circumference; HGS: handgrip strength; ROM: range of motion; FES-I: Falls Efficacy Scale — International.

Of the 765 patients seen during the study period, 255 were invited to participate, of which 79 declined (20 patients feared missing transportation back home) and 32 did not meet the eligibility criteria ( Figure 1 ). Of the 144 remaining participants included in the study, the majority (88.3%) were white and elderly (66.9%), and the mean age was 71.6 (8.30 years), ( Table 1 ). According to BMD results, 33 (34.37%) participants had osteopenia, 61 (64.58%) had osteoporosis, and 1 (1%) had a normal BMD ( Table 2 ).

**Table 1:** Sociodemographic and clinical characteristics and history of falls in the study participants

		Mean ± SD	N (%)
Anthropometric and clinical characteristics			
Age (years)		71.6 ± 8.2	
	<65 years	60.10 ± 7.10	41 (28.1%)
	≥65 years	73.6 ± 7.10	105 (71.9%)
Race			
	White		41 (88.3%)
	Black		4 (11.7%)
Comorbidities			
	Hypertension		17 (11.7%)
	Dyslipidemia		14 (9.7%)
	Smoking		11 (7.6%)
	Diabetes		9 (6.2%)
	Osteoarthritis		6 (4.1%)
BMI (kg/m²)		26.8 ± 5.3	1 (0.7%)
	Malnutrition	21.9 ± 0.7	24 (16.7%)
	Normal (23 < BMI < 28 kg/m^2^)	24.9 ± 1.5	29 (20.0%)
	Pre-obesity (28 < BMI < 30 kg/m^2^)	29.2 ± 0.7	10 (6.9%)
	Obesity (BMI > 30 kg/m^2^)	34.21 ± 4.1	21 (14.5%)
MMSE (score)		25.6 ± 4.1	
WC (cm)		93.7 ± 12.4	
WC > 88 cm		100.67 ± 8.4	59 (40.7%)
History of falls			
Total falls		133	
Number of falls per year (mean)		1.7 ± 1.6	
Non-fallers		0.0	60 (49.5%)
Fallers		1.0 ± 0.0	35 (28.9%)
Recurrent fallers		3.1 ± 1.6	26 (21.5%)

SD: standard deviation; N: absolute frequency; %: relative frequency; BMI: body mass index; kg: kilogram; m: meter; MMSE: Mini-Mental State Examination; WC: waist circumference; cm: centimeter.

**Table 2:** Intrinsic and extrinsic risk factors for falls

Intrinsic Factors		Mean ± SD (n%)
BMD		
	Normal	1 (1%)
	Osteopenia	33 (34.4%)
	Osteoporosis	61 (64.6%)
TUGT (s)		11.7 ± 5.0
	Reduced mobility and risk of falling n (%)	88 (60.7%)
SST (s)		1.0 ± 5.7
	Reduced strength and power n (%)	69 (47.6%)
	Risk of falling (RV:15 s)	40 (27.%)
GS (m/s)		0.82 ± 0.7
	Low (≤0.8 m/s)	18 (1.4%)
	Partially low (>0.6 and ≤1 m/s)	64 (44.1%)
HGS (kgf), RV ≥ 16 kgf		20.0 ± 49
	Low	23 (15.9%)
ROM dorsiflexors (°)		14.7 ± 4.3
	Low (<26 ± 6.3°)	32 (22.1%)
ROM plantar flexors (°)		49.0 ± 1.5
	Low (<57 ± 7.2°)	20 (13.8%)
Calf circumference (cm)		35.6 ± 4.1
	Decreased (<31 cm)	12 (8.3%)
FES-I		28.8 ± 9.8
	Sporadic fall (>23)	38 (26.2%)
	Recurrent fall (>31)	35 (24.1%)
	**Extrinsic factors**	
	**Risk factors**	
	Stairs	54 (37.2%)
	Loose mats	49 (33.8%)
	Slippery bathroom floor	47 (32.4%)
	Loose pets	38 (26.2%)
	Uneven surface	36 (24.8%)
	Slippery surface	25 (1.2%)
	Ramps	24 (16.6%)
	Dim lighting	19 (13.1%)
	Obstacles and tripping hazards	14 (9.7%)
	Missing tiles	7 (4.8%)
	Loose cables or wires	3 (2.1%)
	**Safety factors**	**n (%)**
	Appropriate bed height	33 (22.8%)
	Grab bars in the bathroom	27 (18.6%)
	Stair handrails	22 (15.2%)
	Non-slip carpets	20 (13.8%)
	Anti-slip adhesive on stairs	16 (11.0%)
	Highest chair	17 (11.7%)
	Ramps handrails	8 (5.5%)
	Anti-slippery adhesive on ramps	6 (4.1%)
	Tall toilet seat	6 (4.1%)

TUGT: Timed Up and Go Test; s: seconds; TUGT normal values by age: 60-69 years: 8.1 seconds (s), 70-79 years: 9.2 s, ≥ 80 or more: 11.3 s; CC: calf circumference; cm: centimeters; RV: reference value; GS: gait speed; m: meters; HGS: handgrip strength; kgf: kilogram-force; SST: sit and stand; TUGT: Timed Up and Go Test; s: seconds, TUGT normal values by SST normal values by age: 60-69 years: 11.4 s, 70-79 years: 12.6 s, 80-89 years: 12.7 s; ROM: range of motion; FES-I: Falls Efficacy Scale – International.

Table 1 shows the participants’ sociodemographic and clinical aspects and the history of falls. The mean BMI (26.8 [5.3] kg/m²) was normal, with 6.9% of patients classified with pre-obesity and 14.5% with obesity. The mean WC (93.6 [12 .5] cm) was increased. We observed preserved participants’ cognitive status using the MMSE test. Based on the frequency of falls, we classified the majority as NFG (n = 66, 49.5%), followed by FG (n = 38, 28.9%) and RFG (n = 29, 21.5%). The number of falls was high, with 133 reported, the majority by RFG participants. Age, anthropometric measurements, and MMSE scores were similar between the 3 groups. All groups had normal muscle mass values measured by CC, but physical performance was low in all groups. The RFG showed worse performance than NFG in GS (partially low in 69.8% of RFG vs. 45% of NFG, *P* = .017). The 3 groups had similar and normal HGS, but the inferior members’ strength evaluated by the SST was low in all groups and lowest in RFG ( *P* = .029). The ankle mobility decreased in all groups, and the ROM in the dorsiflexors was about 50% below the minimum normal values, with an average of 70% of patients having decreased ROM ( Table 3 ).

**Table 3:** Comparison of intrinsic factors for falls between study groups

Variable			NFG	FG	RFG	P
			Mean ± SD	Mean ± SD	Mean ± SD	
Age (years)			71.0 ± 8.3	71.2 ± 8.4	74.3 ± 7.8	0.202 +
BMI (kg/m²)			26.5 ± 5.1	25.7 ± 4.1	29.6 ± 7.0	0.210
WC (cm)			94.1 ± 11.6	93.2 ± 13.2	92.9 ± 14.0	0.908 #
MMSE			25.6 ± 4.0	26.0 ± 4.2	26.2 ± 3.3	0.845 #
BMD (T-score SD)						
	Spine		-2.3 ± 1.3	-2.3 ± 1.1	-2.1 ± 1.1	0.656
	Femoral neck		-2.0 ± 1.1	-1.9 ± 0.76	-1.7 ± 0.6	0.079
	Total hip		-1.4 ± 1.2	-1.0 ± 1.0	-1.2 ± 1.0	0.166
CC (cm)			35.4 ± 4.4	36.1 ± 4.2	35.3 ± 3.5	0.626 #
	Reduced, n (%)		7 (11.1%)	3 (7.7%)	2 (9.1%)	0.847
Performance						
	TUGT (s)		11.5 ± 4.8	11.6 ± 5.0	12.4 ± 5.6	0.983 #
		Low, n (%)	41 (77.4%)	30 (73.2%)	17 (70.8%)	0.804
	GS (m/s)		0.81 ± 0.21	0.85 ± 0.27	0.82 ± 0.40	0.588 #
		Low, n (%)	7 (13.2%)	4 (12.9%)	7 (35.0%)	0.017*
		Partially low, n (%)	37 (69.8%)	18 (58.1%)	9 (45.0%)	**0.029**+ *
		Normal, n (%)	9 (17.0%)	7 (22.6%)	1 (5.0%)	0.156
		Fast, n (%)	0 (0%)	2 (6.5%)	3 (15.0%)	
Strength						
	HGS (kgf)		20.0 ± 4.7	19.6 ± 5.3	21.0 ± 5.01	0.326 #
		Low, n (%)	10 (16.1%)	9 (22.5%)	4 (17.4%)	0.713
	SST (s)		14.9 ± 5.2	13.5 ± 3.6	17.7 ± 8.7	**0.029**+ *
		High fall risk, n (%)	24 (38.7%)	7 (21.2%)	9 (42.9%)	**0.156**
		Reduced strength, n (%)	38 (70.4%)	17 (53.1%)	14 (73.7%)	**0.191**
Ankle mobility (ROM)						
	Dorsiflexors (°)		14.9 ± 4.1	14.1 ± 4.7	15.1 ± 4.5	0.822 #
		Low, n (%)	16 (80.0%)	9 (75.0%)	7 (70.0%)	0.827
	Plantar flexors (°)		50.8 ± 13.3	50.7 ± 11.0	42.4 ± 11.4	0.211 #
		Low, n (%)	9 (45.0%)	5 (41.7%)	6 (60.0%)	0.657
	FES-I		29.9 ± 11.7	27.9 ± 6.8	27.4 ± 8.5	0.762 #
		Sporadic falls, n (%)	18 (32.7%)	13 (38.2%)	7 (31.8%)	0.985
		Recurrent falls, n (%)	18 (32.7%)	10 (29.4%)	7 (31.8%)	

NFG: non-faller group; FG: faller group; RFG: recurrent faller group; SD: standard deviation; N: absolute frequency; %: relative frequency; BMI: body mass index; kg: kilogram; m: meter; WC: waist circumference; cm: centimeter; MMSE: Mini-Mental State Examination; TUGT: Timed Up and Go Test; s: second; CC: calf circumference; GS: gait speed; HGS: handgrip strength; kgf: kilogram-force; SST: Sit-to-Stand Test; ROM: range of motion; FES-I: Falls Efficacy Scale – International.

+ one-way ANOVA;

# Kruskal-Wallis test; Bonferroni *post hoc* test between groups FG and RFG;

* p < 0.05, chi-square test.

The number of safety factors against falls tended to be higher in the RFG ( *P* = .052). Extrinsic factors related to the home environment were similar between groups, except for antislip adhesives on stairs, which were more frequent in RFG (P = .009) ( Supplementary Table 1 ).

**Supplementary Table 1:** Comparison of extrinsic factors for falls between study groups

Risk factors	NFG (n = 39)	FG (n = 36)	RFG (n = 21)	χ^2^
				p value
Stairs	20 (51.3%)	22 (61.1%)	12 (57.1%)	0.689
Ramps	13 (33.3%)	9 (25.0%)	2 (9.5%)	0.127
Uneven surface	11 (28.2%)	13 (36.1%)	12 (57.1%)	0.085
Loose mats	19 (48.7%)	18 (50.0%)	12 (57.1%)	0.814
Missing tiles	2 (5.1%)	4 (11.1%)	1 (4.8%)	0.536
Loose cables or wires	2 (5.1%)	1 (5.8%)	0 (0%)	0.546
Slippery surface	12 (30.8%)	7 (19.4%)	6 (28.6%)	0.513
Dim lighting	10 (25.6%)	5 (13.9%)	4 (19.0%)	0.441
Slippery bathroom floor	21 (53.8%)	16 (44.4%)	10 (47.6%)	0.711
Loose pets	17 (43.6%)	13 (36.1%)	8 (38.1%)	0.793
Obstacles and tripping hazards	6 (15.4%)	6 (16.7%)	2 (9.5%)	0.749
Total (mean ± SD)	3.1 ± 2.1	3.3 ± 1.6	3.7 ± 1.5	0.280
**Safety factors**				
Anti-slip adhesive on stairs	3 (7.7%)	5 (13.9%)	8 (38.1%)	0.009
Non-slip carpets	5 (12.8%)	11 (30.6%)	4 (19.0%)	0.163
Anti-slip adhesive on ramps	3 (7.7%)	2 (5.6%)	1 (4.8%)	0.884
Grab bars in the bathroom	7 (17.9%)	12 (33.3%)	8 (38.1%)	0.173
Stair handrails	5 (12.8%)	10 (27.8%)	7 (33.3%)	0.134
Ramp handrails	3 (7.7%)	4 (11.1%)	1 (4.8%)	0.692
Appropriate bed height	10 (25.6%)	16 (44.4%)	7 (33.3%)	0.229
Tallest chair at home	8 (20.5%)	8 (22.2%)	1 (4.8%)	0.209
Tallest toilet seat at home	2 (5.1%)	3 (8.3%)	1 (4.8%)	0.807
Total (mean ± SD)	1.4 ±1.5	1.3 ± 1.0	2.5 ± 1.9	0.052

NFG: non-fallers group; FG: fallers group; RFG: recurrent fallers group; χ^2^: chi-square test.

The frequency of falls was associated with GS ( *P* = .009), uneven floor surface (P = .042), stair handrails ( *P* = .048), and total safety factors (r = 0.2588, *P* = .010) and tended to be associated with the presence of ramps ( *P* = .051) and grab bars in the bathroom ( *P* = .061).

The multivariate analysis considered as a dependent variable the number of falls, and the independent variables of race, GS classification, presence of ramps, uneven surface, antiadhesive on stairs, stair handrails, grab bars in the bathroom, and total safety factors showed that the presence of ramps (RR 0.48, 95% CI, 0.26-0.87, *P* = .015), uneven surface (RR 1.6, 95% CI, 1.05-2.43, *P* = .028), and antislippery adhesive on stairs RR 2.75 (95% CI, 1.77-4.28, P < .001) emerged as significantly associated with the number of falls.

## DISCUSSION

This study showed that in a group of elderly women in the community undergoing treatment for osteoporosis, the extensive evaluation of intrinsic factors for falls showed that physical performance was compromised, with strength of lower limbs and ankle mobility severely affected in all groups, leading to a high risk of falls. But only the SST was able to identify participants with a higher risk of falls (RFG). Given that all groups had intrinsic factors compromised, the difference in the number of falls was possibly underestimated by some patients due a memory bias of falls, as seen in other studies ( 21 , 22 ).

Another aspect identified in this study was the vulnerability of elderly women undergoing treatment to new episodes of falls, which had already been observed in another study ( 23 ).

Falls are multifactorial events that occur in 35%- 40% of the elderly population and may reach 78% when 4 or more risk factors are identified ( 24 , 25 ). The prevalence of falls in the study population was 50%, which is slightly higher than that reported in the literature. Each participant had, on average, 3-4 risk factors for falls and only a few protective factors against falls (average of two). In line with the literature, the presence of multiple intrinsic factors for falls (decreased mobility, ankle amplitude, GS, and muscle strength and power), highlight the greater risk of falls and fractures and decreased musculoskeletal function and mobility among women with low BMD ( 26 ).

Gait speed scores were associated with the number of falls and with worse functional performance and lower limb strength in the RFG. These results disagree with a study with elderly women with osteoporosis whereas no difference between the frequency of falls and functional physical performance were found ( 27 ).

Ankle movements are essential to trigger postural balance strategies and prevent stumbling ( 27 ). We found reduced amplitude of ankle dorsiflexor and plantar-flexor muscles in all groups. The reduced amplitude of movement in these muscles can influence gait performance in the elderly since they generate energy in the ankle ( 27 , 28 ) also, they have important function in the reactive response of balance recovery after a slipping or in situations of balance disturbance. The muscular synergy of the lower limbs is essential for the resumption of postural control, and the thigh extensor muscles play a very important role in recurrent falls. When the reduced ankle ROM is associated to lower limb strength, the risk of falls increases ( 29 ). Although the ankle ROM do not differentiate groups, studies that have correlated the ankle function of elderly women undergoing osteoporosis treatment with falls are scarce and should be subject of new studies.

The cognitive assessment, as well as the HGS results and CC measurements, were adequate in all groups. These are considered independent risk factors for increased fear of falling and deterioration of quality of life ( 29 ). We observed that 25% of the patients had a fear of falling, which was compatible with the occurrence of sporadic or recurrent falls, as previously observed in patients with kyphosis and osteoporosis ( 30 ).

Among the extrinsic factors for falls, there was a high prevalence of risk factors with only a few safety factors against falls reported, mainly in the RFG. Only 16 (11%) patients had a safety device on the stairs. The most frequently reported safety factor was an appropriate bed height, as previously described ( 30 , 31 ). The number of falls was associated with several extrinsic factors, although the presence of ramps, uneven surface, and adhesive on the stairs were the factors associated with the highest number of falls. These findings are aligned with the results of a study in elderly women that reported the presence of extrinsic risks in the home environment ( 30 , 31 ).

The limitations of this study include the cross-sectional design and the absence of a control group, blind assessment, and information regarding the use of medications by the participants. Also, should be taken into account the possibility of memory bias when considering the number of falls. A positive aspect of the study was the broad screening of risk factors for falls using low-cost instruments that managed to capture factors associated with falls.

In conclusion, this study showed that patients receiving treatment for osteoporosis are influenced by intrinsic and extrinsic factors for falls. Lower limb strength and power discriminated participants at a higher risk of falls, while extrinsic factors varied, and only uneven floor and anti-slippery adhesives on stairs were associated with an increased frequency of falls.

## References

[1] Melton LJ (1995). How many women have osteoporosis now?. J Bone Miner Res.

[2] Viswanathan M, Reddy S, Berkman N, Cullen K, Middleton JC, Nicholson WK (2018). Screening to Prevent Osteoporotic Fractures: Updated Evidence Report and Systematic Review for the US Preventive Services Task Force. JAMA.

[3] Hopkins RB, Burke N, Von Keyserlingk C, Leslie WD, Morin SN, Adachi JD (2016). The current economic burden of illness of osteoporosis in Canada. Osteoporos Int.

[4] Clemson L, Mackenzie L, Ballinger C, Close JC, Cumming RG (2008). Environmental interventions to prevent falls in community-dwelling older people: a meta-analysis of randomized trials. J Aging Health.

[5] Phelan EA, Mahoney JE, Voit JC, Stevens JA (2015). Assessment and management of fall risk in primary care settings. Med Clin North Am.

[6] Trombetti A, Reid KF, Hars M, Herrmann FR, Pasha E, Phillips EM (2016). Age-associated declines in muscle mass, strength, power, and physical performance: impact on fear of falling and quality of life. Osteoporos Int.

[7] Moreira NB, Rodacki ALF, Pereira G, Bento PCB (2018). Does functional capacity, fall risk awareness and physical activity level predict falls in older adults in different age groups?. Arch Gerontol Geriatr.

[8] Arnold CM, Busch AJ, Schachter CL, Harrison L, Olszynski W (2005). The relationship of intrinsic fall risk factors to a recent history of falling in older women with osteoporosis. J Orthop Sports Phys Ther.

[9] Clemson L, Mackenzie L, Ballinger C, Close JC, Cumming RG (2008). Environmental interventions to prevent falls in community-dwelling older people: a meta-analysis of randomized trials. J Aging Health.

[10] Rossetin LL, Rodrigues EV, Gallo LH, Macedo DS, Schieferdecker MEM, Pintarelli VL (2016). Indicators of sarcopenia and their relation to intrinsic and extrinsic factors relating to falls among active elderly women. Rev Bras Geriatr Gerontol.

[11] Wang X, Ma Y, Wang J, Han P, Dong R, Kang L (2016). Mobility and Muscle Strength Together are More Strongly Correlated with Falls in Suburb-Dwelling Older Chinese. Sci Rep.

[12] Cederholm T, Bosaeus I, Barazzoni R, Bauer J, Van Gossum A, Klek S (2015). Diagnostic criteria for malnutrition – An ESPEN Consensus Statement. Clin Nutr.

[13] Lebrão ML, Duarte YA (2003). SABE-health, well-being and aging, the Sabe Project in the city of São Paulo: an approach that begins. SABE-health, well-being and aging, the Sabe Project in the city of São Paulo: an initial approach.

[14] Lebrão ML, Duarte YAO, SABE – Health, Wellness and Aging (2003). The Sabe Project in the city of São Paulo: an initial approach.

[15] Lourenço RA, Veras RP (2006). Mini-Mental State Examination: psychometric characteristics in elderly outpatients. Rev Saude Publica.

[16] Podsiadlo D, Richardson S (1991). The timed “Up & Go”: a test of basic functional mobility for frail elderly persons. J Am Geriatr Soc.

[17] Cruz-Jentoft AJ, Bahat G, Bauer J, Boirie Y, Bruyère O, Cederholm T, Writing Group for the European Working Group on Sarcopenia in Older People 2 (EWGSOP2), and the Extended Group for EWGSOP2 (2019). Sarcopenia: revised European consensus on definition and diagnosis. Age Ageing.

[18] Bohannon RW (2006). Reference values for the timed up and go test: a descriptive meta-analysis. J Geriatr Phys Ther.

[19] McKay MJ, Baldwin JN, Ferreira P, Simic M, Vanicek N, Burns J, 1000 Norms Project Consortium (2017). Normative reference values for strength and flexibility of 1,000 children and adults. Neurology.

[20] Camargos FF, Dias RC, Dias JM, Freire MT (2010). Cross-cultural adaptation and evaluation of the psychometric properties of the Falls Efficacy Scale-International Among Elderly Brazilians (FES-I-BRAZIL). Rev Bras Fisioter.

[21] Liu IT, Liang FW, Wang ST, Chang CM, Lu TH, Wu CH (2021). The effects of falls on the prediction of osteoporotic fractures: epidemiological cohort study. Arch Osteoporos.

[22] Hauer K, Lamb SE, Jorstad EC, Todd C, Becker C, PROFANE-Group (2006). Systematic review of definitions and methods of measuring falls in randomised controlled fall prevention trials. Age Ageing.

[23] Sanders KM, Hayles AL, Kotowicz MA, Nicholson GC (2009). Monitoring falls in cohort studies of community-dwelling older women. J Am Geriatr Soc.

[24] Trombetti A, Reid KF, Hars M, Herrmann FR, Pasha E, Phillips EM, Fielding RA (2016). Age-associated declines in muscle mass, strength, power, and physical performance: impact on fear of falling and quality of life. Osteoporos Int.

[25] Panel on Prevention of Falls in Older Persons, American Geriatrics Society and British Geriatrics Society (2011). Summary of the Updated American Geriatrics Society/British Geriatrics Society clinical practice guideline for prevention of falls in older persons. J Am Geriatr Soc.

[26] Cöster ME, Karlsson M, Ohlsson C, Mellström D, Lorentzon M, Ribom E (2020). Physical function tests predict incident falls: A prospective study of 2969 men in the Swedish Osteoporotic Fractures in Men study. Scand J Public Health.

[27] Stief F, Schäfer A, Vogt L, Kirchner M, Hübscher M, Thiel C (2016). Differences in Gait Performance, Quadriceps Strength, and Physical Activity Between Fallers and Nonfallers in Women with Osteoporosis. J Aging Phys Act.

[28] Tinetti ME, Kumar C (2010). The patient who falls: “It's always a trade-off”. JAMA.

[29] Hernández-Guillén D, Tolsada-Velasco C, Roig-Casasús S, Costa-Moreno E, Borja-de-Fuentes I, Blasco JM (2021). Association ankle function and balance in community-dwelling older adults. PLoS One.

[30] Cöster ME, Karlsson M, Ohlsson C, Mellström D, Lorentzon M, Ribom E (2020). Physical function tests predict incident falls: A prospective study of 2969 men in the Swedish Osteoporotic Fractures in Men study. Scand J Public Health.

[31] Pinheiro MM, Ciconelli RM, Martini LA, Ferraz MB (2009). Clinical risk factors for osteoporotic fractures in Brazilian women and men: the Brazilian Osteoporosis Study (BRAZOS). Osteoporos Int.

